# (±)-1-{8′-(*tert*-Butyl­diphenyl­silyloxy­meth­yl)-1′,7′-dioxaspiro­[5.5]undecan-2′-yl}uridine

**DOI:** 10.1107/S1600536808006727

**Published:** 2008-03-14

**Authors:** Ka Wai Choi, Margaret A. Brimble, Tania Groutso

**Affiliations:** a Department of Chemistry, Univerisity of Auckland, Private Bag 92019, Auckland, New Zealand

## Abstract

The crystal structure of the title compound, C_30_H_38_N_2_O_5_Si, has been investigated to establish the relative stereochemistry at the spiro ring junction and the two anomeric centres. Each of the O atoms in the tetra­hydro­pyran rings adopts an axial position on the neighbouring ring. This *bis­*-diaxial conformation is adopted, thus gaining maximum stablization from the anomeric effect. The silyl-protected hydroxy­methyl and uracil substituents adopt equatorial positions on their associated tetra­hydro­pyran rings, thereby minimizing unfavourable steric inter­actions. The dimeric (2′R*,6′R*,8′R*)- and (2′S*,6′S*,8′S*)-uridine units are connected to each other across crystallographic inversion centres *via* inter­molecular N—H⋯O hydrogen bonds.

## Related literature

For related literature, see: Mead & Zemribo (1996 ▶); Brimble *et al.* (1998 ▶, 2004 ▶).
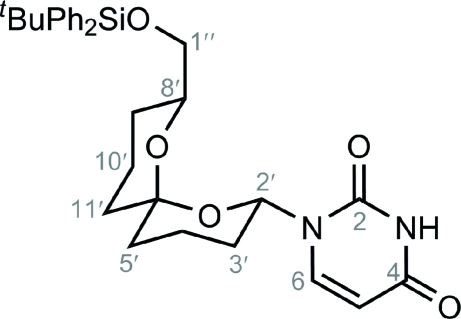


## Experimental

### 

#### Crystal data

C_30_H_38_N_2_O_5_Si
*M* *_r_* = 534.71
Monoclinic, 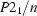

*a* = 14.7960 (2) Å
*b* = 12.5092 (2) Å
*c* = 15.0935 (1) Å
β = 99.420 (1)°
*V* = 2755.93 (6) Å^3^
*Z* = 4
Mo *K*α radiation
μ = 0.13 mm^−1^
*T* = 293 (2) K
0.32 × 0.26 × 0.12 mm

#### Data collection

Siemens SMART CCD diffractometer
Absorption correction: multi-scan (*SADABS*; Sheldrick, 1996 ▶) *T*_min_ = 0.960, *T*_max_ = 0.985
15898 measured reflections
5612 independent reflections
4327 reflections with *I* > 2σ(*I*)
*R*_int_ = 0.044

#### Refinement

*R*[*F* ^2^ > 2σ(*F* ^2^)] = 0.052
*wR*(*F* ^2^) = 0.111
*S* = 1.09
5612 reflections
343 parameters
H-atom parameters constrained
Δρ_max_ = 0.30 e Å^−3^
Δρ_min_ = −0.34 e Å^−3^

### 

Data collection: *SMART* (Siemens, 1995 ▶); cell refinement: *SAINT* (Siemens, 1995 ▶); data reduction: *SAINT*; program(s) used to solve structure: *SHELXS97* (Sheldrick, 2008 ▶); program(s) used to refine structure: *SHELXL97* (Sheldrick, 2008 ▶); molecular graphics: *ORTEPIII* (Burnett & Johnson, 1996 ▶) and *Mercury* (Macrae *et al.*, 2006 ▶); software used to prepare material for publication: *SHELXTL* (Sheldrick, 2008 ▶) and *publCIF* (Westrip, 2008 ▶).

## Supplementary Material

Crystal structure: contains datablocks I, global. DOI: 10.1107/S1600536808006727/si2075sup1.cif

Structure factors: contains datablocks I. DOI: 10.1107/S1600536808006727/si2075Isup2.hkl

Additional supplementary materials:  crystallographic information; 3D view; checkCIF report

## Figures and Tables

**Table 1:** Hydrogen-bond geometry (Å, °)

*D*—H⋯*A*	*D*—H	H⋯*A*	*D*⋯*A*	*D*—H⋯*A*
N3—H3*A*⋯O4^i^	0.86	2.03	2.873 (2)	166

Symmetry code: (i) 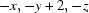.

**Fig. 1.**
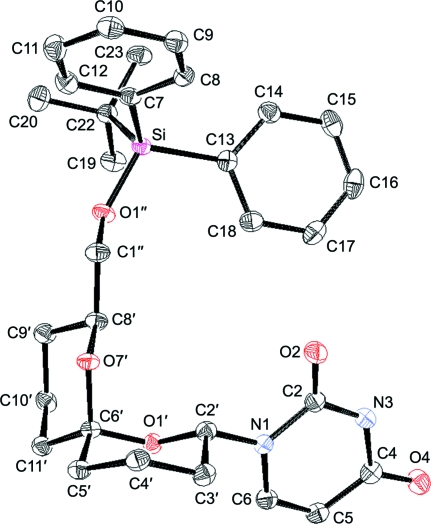
The molecular structure and atom numbering scheme of (2'S*,6'S*,8'S*)-uridine with displacement ellipsoids drawn at the 50% probability level.

**Fig. 2.**
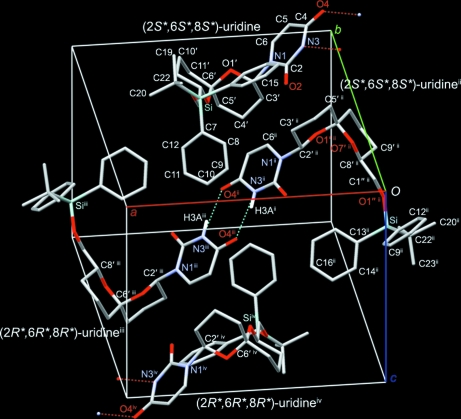
Molecular packing of racemic uridine units. The dimeric units of opposite chirality, (2'R*,6R'*,8'R*)- and (2'S*,6'S*,8'S*)-uridines are connected to each other by intermolecular hydrogen bonds. Dashed lines represent hydrogen bonds. Most hydrogen atoms that are not involved in hydrogen bonding, have been omitted for clarity. The origin of the unit cell is labelled as O while cell axes are labelled as a (red), b (green) and c (blue), respectively. [Symmetry code: (ii) 1/2 - x, -1/2 + y, 1/2 - z; (iii) 1/2 + x, 3/2 - y, 1/2 + z; (iv) -x + 1, -y + 1, -z + 1.]

## supplementary crystallographic information

### Comment

The title uridine was prepared as a part of study to elaborate 6,6-spiroacetal scaffolds to incorporate a nucleobase at the anomeric position, thus generating a collection of novel hybrid structures. Similar syntheses of 6,6-spiroacetal based molecules in the presence of a Lewis acid and persilylated nucleophile have been reported by Mead & Zemribo (1996) and Brimble *et al.* (1998, 2004).

Figure 1 depicts the structure and atom numbering of the title uridine. The spiroacetal ring system adopts a conformation in which each of the O atoms (O1' and O7') adjacent to the C6' spirocentre adopts an axial position on the neighbouring ring, thus gaining maximum stabilization from the anomeric effect. The silyl-protected hydroxymethyl and uracil substituents adopt equatorial positions on their associated tetrahydropyran rings in order to minimized unfavourable steric interactions.

Figure 2 depicts molecular packing of racemic uridine units. The dimeric (*2'R*,6'R*,8'R**) and (*2'S*,6'S*,8'S**)-uridine units are connected to each other by the crystallographic inversion centres *via* intermolecular N3–H3A···O4 hydrogen bonds (Table 1).

### Experimental

To a suspension of uracil (6.95 mg, 61.9 µmol) in hexamethyldisilazane (0.5 ml) under an atmosphere of argon was added ammonium sulfate (2 crystals) and the mixture was heated to reflux until the white solid dissolved. After 3 h, the mixture was concentrated *in vacuo* to a thick yellow oil. 8-(*tert*-Butyldiphenylsilyloxymethyl)-2-acetoxy-1,7-dioxaspiro[5.5] undecane (18.8 mg, 38.9 µmol) in CH_2_Cl_2_ (1.0 ml) was transferred to the yellow oil *via* cannula. Freshly prepared TMSOTf solution (95.4 µ*L*, 66.8 µmol, 0.70 mol *L*^-1^ in CH_2_Cl_2_) was added dropwise. After 3 h, saturated NaHCO_3_ solution (2 ml) and CH_2_Cl_2_ (2 ml) were added and the mixture was stirred for 15 min. The aqueous phase was extracted with CH_2_Cl_2_ (3 *x* 4 ml). The combined organic extracts were dried over MgSO_4_ and concentrated *in vacuo*. Purification by flash chromatography using hexane–EtOAc (19:1 to 7:3) as eluent yielded the title compound (7.50 mg, 36%) as a pale-yellow powder. Recrystallization from hexane–CH_2_Cl_2_ afforded pale yellow needles.

HRMS (FAB): found MH^+^, 535.2633, C_30_H_39_N_2_O_5_Si requires 535.2628.

*ν*_max_ (film)/cm^-1^: 3376 (N–H), 2919 (C–H), 1689 (C=O), 1668 (C=O), 1456, 1377, 1267 (C–O), 1103 (C–O), 982, 699.

*δ*_H_ (300 MHz; CDCl_3_): 1.07 (9 H, s, OSiPh_2_*^t^*Bu), 1.31–1.38 (1 H, m, H9'A), 1.41–1.51 (3 H, m, H3'A, H5'A and H11'A), 1.56–1.76 (5 H, m, H4'A, H5'B, H9'B, H10'A and H11'B), 1.80–1.94 (2 H, m, H3'B and H10'B), 2.07–2.16 (1 H, m, H4'B), 3.63 (1 H, dd, *J*_AB_ 10.4 and *J*_H1''A,8'_ 4.5, H1''A), 3.72 (1 H, dd, *J*_AB_ 10.4 and *J*_H1''B,8'_ 5.3, H1''B), 3.82–3.89 (1 H, m, H8'), 5.73 (1 H, d, *J*_5,6_ 8.2, H5), 5.94 (1 H, dd, *J*_2'ax,3'ax_ 11.1 and *J*_2'ax,3'eq_ 2.5, H2'_ax_), 7.33–7.42 (6 H, m, Ph), 7.46 (1 H, d, *J*_6,5_ 8.2, H6), 7.70–7.76 (4 H, m, Ph), 8.17 (1 H, br s, NH).

*δ*_C_ (75 MHz; CDCl_3_): 17.9 (CH_2_, C4'), 18.0 (CH_2_, C10'), 19.3 (C, OSiPh_2_*^t^*Bu), 26.5 (CH_2_, C9'), 26.8 (CH_3_, OSiPh_2_*^t^*Bu), 30.3 (CH_2_, C3'), 34.7 (CH_2_, C5'), 34.8 (CH_2_, C11'), 67.0 (CH_2_, C1''), 70.7 (CH, C8'), 76.8 (CH, C2'), 99.1 (C, C6'), 102.1 (CH, C5), 127.6 (CH, Ph), 129.5 (CH, Ph), 129.5 (CH, Ph), 133.8 (C, Ph), 135.7 (CH, Ph), 135.7 (CH, Ph), 140.3 (CH, C6), 149.7 (C, C2), 162.8 (C, C4).

*m/z* (FAB): 535 (MH^+^, 3%), 477 (*M* – *^t^*Bu, 11), 457 (*M* – Ph, 3), 423 (C_26_H_35_O_3_Si, 19), 239 (SiPh_2_*^t^*Bu, 8), 199 (35), 197 (35), 135 (100), 105 (32), 91 (73).

### Refinement

H atoms were placed in calculated positions and were refined using a riding model (C—H = 0.93 or 0.97 Å), with *U *_iso_(H) = 1.2 or 1.5 times *U*_eq_(C).

### Crystal data

**Table:**

C_30_H_38_N_2_O_5_Si	*D*_x_ = 1.289 Mg m^−^^3^
*M**_r_* = 534.71	Melting point: 482.4(9) K
Monoclinic, *P*2_1_/*n*	Mo *K*α radiation λ = 0.71073 Å
*a* = 14.7960 (2) Å	Cell parameters from 5612 reflections
*b* = 12.5092 (2) Å	θ = 1.8–26.4º
*c* = 15.0935 (1) Å	µ = 0.13 mm^−^^1^
β = 99.420 (1)º	*T* = 293 (2) K
*V* = 2755.93 (6) Å^3^	Needle, pale yellow
*Z* = 4	0.32 × 0.26 × 0.12 mm
*F*_000_ = 1144	

### Data collection

**Table:**

Siemens SMART CCD diffractometer	5612 independent reflections
Radiation source: fine-focus sealed tube	4327 reflections with *I* > 2σ(*I*)
Monochromator: graphite	*R*_int_ = 0.044
*T* = 293(2) K	θ_max_ = 26.4º
area–detector ω scans	θ_min_ = 1.8º
Absorption correction: multi-scan(SADABS; Sheldrick, 1996)	*h* = −14→18
*T*_min_ = 0.960, *T*_max_ = 0.985	*k* = −15→12
15898 measured reflections	*l* = −18→18

### Refinement

**Table:**

Refinement on *F*^2^	Secondary atom site location: difference Fourier map
Least-squares matrix: full	Hydrogen site location: inferred from neighbouring sites
*R*[*F*^2^ > 2σ(*F*^2^)] = 0.052	H-atom parameters constrained
*wR*(*F*^2^) = 0.111	*w* = 1/[σ^2^(*F*_o_^2^) + (0.0251*P*)^2^ + 2.8069*P*] where *P* = (*F*_o_^2^ + 2*F*_c_^2^)/3
*S* = 1.09	(Δ/σ)_max_ = 0.001
5612 reflections	Δρ_max_ = 0.30 e Å^−^^3^
343 parameters	Δρ_min_ = −0.34 e Å^−^^3^
Primary atom site location: structure-invariant direct methods	Extinction correction: none

### Special details

**Table:**

Geometry. All e.s.d.'s (except the e.s.d. in the dihedral angle between two l.s. planes) are estimated using the full covariance matrix. The cell e.s.d.'s are taken into account individually in the estimation of e.s.d.'s in distances, angles and torsion angles; correlations between e.s.d.'s in cell parameters are only used when they are defined by crystal symmetry. An approximate (isotropic) treatment of cell e.s.d.'s is used for estimating e.s.d.'s involving l.s. planes.
Refinement. Refinement of *F*^2^ against ALL reflections. The weighted *R*-factor *wR* and goodness of fit *S* are based on *F*^2^, conventional *R*-factors *R* are based on *F*, with *F* set to zero for negative *F*^2^. The threshold expression of *F*^2^ > 2σ(*F*^2^) is used only for calculating *R*-factors(gt) *etc*. and is not relevant to the choice of reflections for refinement. *R*-factors based on *F*^2^ are statistically about twice as large as those based on *F*, and *R*- factors based on ALL data will be even larger.

### Fractional atomic coordinates and isotropic or equivalent isotropic displacement parameters (Å^2^)

**Table:**

	*x*	*y*	*z*	*U*_iso_*/*U*_eq_	
Si	0.55794 (4)	0.75101 (5)	0.10014 (4)	0.01617 (14)	
O1'	0.36047 (10)	1.12404 (11)	0.28260 (9)	0.0173 (3)	
O1''	0.56790 (11)	0.86029 (11)	0.16209 (10)	0.0199 (3)	
O2	0.19564 (11)	0.89649 (12)	0.17326 (11)	0.0253 (4)	
O4	0.00327 (12)	1.13783 (12)	0.01545 (10)	0.0267 (4)	
N1	0.21693 (12)	1.07393 (14)	0.21025 (12)	0.0179 (4)	
N3	0.10473 (13)	1.02092 (14)	0.09140 (12)	0.0205 (4)	
H3A	0.0807	0.9715	0.0555	0.025*	
C1''	0.53531 (16)	0.87555 (17)	0.24520 (14)	0.0208 (5)	
H1''A	0.5834	0.8591	0.2951	0.025*	
H1''B	0.4837	0.8287	0.2484	0.025*	
C2	0.17425 (15)	0.98991 (17)	0.15908 (14)	0.0188 (5)	
C2'	0.28987 (15)	1.04722 (17)	0.28551 (14)	0.0180 (4)	
H2'A	0.3136	0.9758	0.2758	0.022*	
C3'	0.25589 (15)	1.04928 (18)	0.37497 (14)	0.0204 (5)	
H3'A	0.2081	0.9962	0.3752	0.025*	
H3'B	0.2304	1.1190	0.3844	0.025*	
C4'	0.33596 (15)	1.02537 (18)	0.44990 (14)	0.0197 (5)	
H4'A	0.3162	1.0321	0.5079	0.024*	
H4'B	0.3572	0.9528	0.4440	0.024*	
C4	0.06939 (16)	1.12308 (18)	0.07495 (14)	0.0208 (5)	
C5	0.11736 (15)	1.20464 (18)	0.13209 (14)	0.0204 (5)	
H5A	0.0996	1.2758	0.1242	0.025*	
C5'	0.41357 (15)	1.10398 (17)	0.44375 (14)	0.0195 (5)	
H5'A	0.3947	1.1752	0.4585	0.023*	
H5'B	0.4667	1.0844	0.4873	0.023*	
C6'	0.43971 (15)	1.10504 (16)	0.35013 (14)	0.0174 (4)	
C6	0.18739 (15)	1.17817 (17)	0.19652 (14)	0.0199 (5)	
H6A	0.2169	1.2317	0.2331	0.024*	
O7'	0.47639 (10)	1.00155 (11)	0.33619 (9)	0.0176 (3)	
C7	0.57397 (15)	0.63171 (16)	0.17660 (13)	0.0172 (4)	
C8	0.50484 (15)	0.55495 (17)	0.17660 (14)	0.0189 (5)	
H8A	0.4524	0.5591	0.1334	0.023*	
C8'	0.50635 (15)	0.99104 (17)	0.24987 (14)	0.0185 (5)	
H8'A	0.4542	1.0045	0.2021	0.022*	
C9'	0.58166 (15)	1.07096 (17)	0.24148 (15)	0.0216 (5)	
H9'A	0.6008	1.0634	0.1833	0.026*	
H9'B	0.6343	1.0573	0.2875	0.026*	
C9	0.51259 (16)	0.47299 (17)	0.23927 (14)	0.0210 (5)	
H9A	0.4657	0.4231	0.2374	0.025*	
C10	0.58946 (16)	0.46522 (18)	0.30429 (15)	0.0225 (5)	
H10A	0.5940	0.4110	0.3470	0.027*	
C10'	0.54654 (16)	1.18395 (17)	0.25179 (14)	0.0212 (5)	
H10'A	0.4993	1.2008	0.2012	0.025*	
H10'B	0.5963	1.2346	0.2523	0.025*	
C11'	0.50739 (15)	1.19351 (17)	0.33940 (14)	0.0193 (5)	
H11'A	0.5573	1.1913	0.3898	0.023*	
H11'B	0.4770	1.2621	0.3406	0.023*	
C11	0.66025 (17)	0.53891 (19)	0.30570 (15)	0.0253 (5)	
H11A	0.7128	0.5331	0.3486	0.030*	
C12	0.65222 (16)	0.62106 (18)	0.24300 (15)	0.0233 (5)	
H12A	0.6997	0.6702	0.2449	0.028*	
C13	0.44029 (15)	0.74260 (17)	0.03190 (13)	0.0186 (4)	
C14	0.41471 (16)	0.66001 (18)	−0.03071 (14)	0.0231 (5)	
H14A	0.4575	0.6079	−0.0386	0.028*	
C15	0.32729 (17)	0.65419 (19)	−0.08109 (15)	0.0265 (5)	
H15A	0.3123	0.5998	−0.1229	0.032*	
C16	0.26269 (17)	0.7308 (2)	−0.06812 (15)	0.0286 (6)	
H16A	0.2040	0.7274	−0.1014	0.034*	
C17	0.28483 (16)	0.8121 (2)	−0.00618 (16)	0.0277 (5)	
H17A	0.2409	0.8623	0.0030	0.033*	
C18	0.37312 (16)	0.81829 (18)	0.04222 (15)	0.0232 (5)	
H18A	0.3880	0.8742	0.0826	0.028*	
C19	0.63144 (17)	0.87945 (18)	−0.02047 (15)	0.0259 (5)	
H19A	0.6769	0.8915	−0.0580	0.039*	
H19B	0.6350	0.9350	0.0238	0.039*	
H19C	0.5717	0.8798	−0.0566	0.039*	
C20	0.74439 (16)	0.7706 (2)	0.08381 (16)	0.0269 (5)	
H20A	0.7896	0.7806	0.0456	0.040*	
H20B	0.7549	0.7037	0.1149	0.040*	
H20C	0.7486	0.8278	0.1267	0.040*	
C22	0.64860 (15)	0.77039 (17)	0.02668 (14)	0.0189 (5)	
C23	0.64515 (17)	0.68136 (18)	−0.04386 (15)	0.0257 (5)	
H23A	0.6922	0.6932	−0.0797	0.039*	
H23B	0.5863	0.6818	−0.0818	0.039*	
H23C	0.6548	0.6134	−0.0142	0.039*	

### Atomic displacement parameters (Å^2^)

**Table:**

	*U*^11^	*U*^22^	*U*^33^	*U*^12^	*U*^13^	*U*^23^
Si	0.0173 (3)	0.0145 (3)	0.0176 (3)	−0.0002 (2)	0.0056 (2)	−0.0012 (2)
O1'	0.0150 (8)	0.0180 (8)	0.0192 (7)	−0.0014 (6)	0.0034 (6)	0.0004 (6)
O1''	0.0240 (9)	0.0181 (8)	0.0197 (7)	−0.0022 (6)	0.0095 (7)	−0.0046 (6)
O2	0.0260 (9)	0.0176 (8)	0.0308 (9)	0.0007 (7)	0.0003 (7)	−0.0006 (7)
O4	0.0294 (9)	0.0247 (9)	0.0235 (8)	0.0001 (7)	−0.0029 (7)	0.0028 (7)
O7'	0.0206 (8)	0.0159 (7)	0.0179 (7)	0.0016 (6)	0.0075 (6)	−0.0022 (6)
N1	0.0160 (9)	0.0184 (9)	0.0194 (9)	0.0006 (7)	0.0028 (7)	−0.0002 (7)
N3	0.0227 (10)	0.0192 (9)	0.0193 (9)	−0.0011 (8)	0.0027 (8)	−0.0020 (8)
C1''	0.0256 (12)	0.0199 (11)	0.0183 (10)	0.0031 (9)	0.0076 (9)	−0.0003 (9)
C2	0.0177 (11)	0.0215 (11)	0.0185 (10)	−0.0007 (9)	0.0066 (9)	−0.0003 (9)
C2'	0.0186 (11)	0.0159 (10)	0.0198 (10)	0.0005 (9)	0.0038 (9)	0.0011 (8)
C3'	0.0187 (11)	0.0202 (11)	0.0237 (11)	−0.0013 (9)	0.0076 (9)	0.0020 (9)
C4'	0.0206 (12)	0.0216 (11)	0.0181 (10)	0.0015 (9)	0.0069 (9)	0.0004 (9)
C4	0.0217 (12)	0.0226 (12)	0.0190 (11)	−0.0001 (9)	0.0062 (9)	0.0052 (9)
C5	0.0223 (12)	0.0174 (11)	0.0226 (11)	0.0021 (9)	0.0068 (9)	0.0039 (9)
C5'	0.0217 (12)	0.0193 (11)	0.0185 (10)	0.0024 (9)	0.0058 (9)	−0.0021 (9)
C6'	0.0172 (11)	0.0162 (10)	0.0187 (10)	0.0017 (9)	0.0031 (9)	−0.0016 (8)
C6	0.0208 (12)	0.0169 (11)	0.0231 (11)	−0.0017 (9)	0.0070 (9)	0.0006 (9)
C7	0.0197 (11)	0.0166 (10)	0.0166 (10)	0.0010 (9)	0.0068 (9)	−0.0025 (8)
C8	0.0159 (11)	0.0200 (11)	0.0213 (11)	0.0006 (9)	0.0049 (9)	−0.0013 (9)
C8'	0.0223 (12)	0.0183 (11)	0.0161 (10)	0.0016 (9)	0.0062 (9)	−0.0016 (8)
C9'	0.0205 (12)	0.0216 (11)	0.0243 (11)	−0.0017 (9)	0.0084 (10)	−0.0028 (9)
C9	0.0207 (12)	0.0185 (11)	0.0255 (11)	−0.0028 (9)	0.0086 (10)	−0.0009 (9)
C10	0.0297 (13)	0.0174 (11)	0.0212 (11)	0.0009 (10)	0.0068 (10)	0.0024 (9)
C10'	0.0224 (12)	0.0192 (11)	0.0226 (11)	−0.0058 (9)	0.0057 (9)	−0.0012 (9)
C11'	0.0188 (11)	0.0176 (11)	0.0220 (11)	−0.0016 (9)	0.0047 (9)	−0.0021 (9)
C11	0.0246 (13)	0.0271 (12)	0.0228 (11)	−0.0010 (10)	−0.0006 (10)	0.0015 (10)
C12	0.0248 (13)	0.0211 (11)	0.0243 (11)	−0.0040 (10)	0.0048 (10)	−0.0010 (9)
C13	0.0197 (11)	0.0191 (11)	0.0176 (10)	−0.0015 (9)	0.0046 (9)	0.0028 (9)
C14	0.0244 (13)	0.0227 (12)	0.0235 (11)	−0.0017 (10)	0.0073 (10)	−0.0022 (9)
C15	0.0304 (14)	0.0299 (13)	0.0188 (11)	−0.0105 (11)	0.0032 (10)	−0.0003 (10)
C16	0.0213 (12)	0.0390 (15)	0.0237 (11)	−0.0044 (11)	−0.0017 (10)	0.0105 (11)
C17	0.0215 (13)	0.0299 (13)	0.0311 (13)	0.0053 (10)	0.0030 (10)	0.0044 (11)
C18	0.0259 (13)	0.0207 (11)	0.0235 (11)	0.0020 (10)	0.0052 (10)	0.0021 (9)
C19	0.0322 (14)	0.0227 (12)	0.0254 (12)	−0.0005 (10)	0.0122 (10)	0.0013 (10)
C20	0.0210 (12)	0.0334 (14)	0.0275 (12)	−0.0019 (10)	0.0077 (10)	0.0027 (10)
C22	0.0186 (11)	0.0192 (11)	0.0203 (10)	−0.0003 (9)	0.0074 (9)	−0.0001 (9)
C23	0.0292 (14)	0.0229 (12)	0.0282 (12)	0.0009 (10)	0.0139 (11)	−0.0030 (10)

### Geometric parameters (Å, °)

**Table:**

Si—O1''	1.6491 (15)	C8'—C9'	1.518 (3)
Si—C13	1.875 (2)	C8'—H8'A	0.9800
Si—C7	1.878 (2)	C9'—C10'	1.523 (3)
Si—C22	1.891 (2)	C9'—H9'A	0.9700
O1'—C2'	1.425 (2)	C9'—H9'B	0.9700
O1'—C6'	1.442 (3)	C9—C10	1.379 (3)
O1''—C1''	1.428 (2)	C9—H9A	0.9300
O2—C2	1.220 (3)	C10—C11	1.393 (3)
O4—C4	1.229 (3)	C10—H10A	0.9300
O7'—C6'	1.433 (2)	C10'—C11'	1.533 (3)
O7'—C8'	1.449 (2)	C10'—H10'A	0.9700
N1—C6	1.380 (3)	C10'—H10'B	0.9700
N1—C2	1.393 (3)	C11'—H11'A	0.9700
N1—C2'	1.472 (3)	C11'—H11'B	0.9700
N3—C2	1.381 (3)	C11—C12	1.389 (3)
N3—C4	1.388 (3)	C11—H11A	0.9300
N3—H3A	0.8600	C12—H12A	0.9300
C1''—C8'	1.512 (3)	C13—C18	1.400 (3)
C1''—H1''A	0.9700	C13—C14	1.409 (3)
C1''—H1''B	0.9700	C14—C15	1.391 (3)
C2'—C3'	1.516 (3)	C14—H14A	0.9300
C2'—H2'A	0.9800	C15—C16	1.390 (3)
C3'—C4'	1.528 (3)	C15—H15A	0.9300
C3'—H3'A	0.9700	C16—C17	1.384 (3)
C3'—H3'B	0.9700	C16—H16A	0.9300
C4'—C5'	1.526 (3)	C17—C18	1.391 (3)
C4'—H4'A	0.9700	C17—H17A	0.9300
C4'—H4'B	0.9700	C18—H18A	0.9300
C4—C5	1.445 (3)	C19—C22	1.540 (3)
C5—C6	1.341 (3)	C19—H19A	0.9600
C5—H5A	0.9300	C19—H19B	0.9600
C5'—C6'	1.525 (3)	C19—H19C	0.9600
C5'—H5'A	0.9700	C20—C22	1.534 (3)
C5'—H5'B	0.9700	C20—H20A	0.9600
C6'—C11'	1.519 (3)	C20—H20B	0.9600
C6—H6A	0.9300	C20—H20C	0.9600
C7—C8	1.403 (3)	C22—C23	1.536 (3)
C7—C12	1.408 (3)	C23—H23A	0.9600
C8—C9	1.387 (3)	C23—H23B	0.9600
C8—H8A	0.9300	C23—H23C	0.9600
			
O1''—Si—C13	110.29 (9)	C9'—C8'—H8'A	109.0
O1''—Si—C7	108.65 (8)	C8'—C9'—C10'	109.60 (18)
C13—Si—C7	107.79 (10)	C8'—C9'—H9'A	109.8
O1''—Si—C22	102.72 (9)	C10'—C9'—H9'A	109.8
C13—Si—C22	111.66 (9)	C8'—C9'—H9'B	109.8
C7—Si—C22	115.59 (10)	C10'—C9'—H9'B	109.8
C2'—O1'—C6'	112.48 (15)	H9'A—C9'—H9'B	108.2
C1''—O1''—Si	126.65 (13)	C10—C9—C8	120.3 (2)
C6'—O7'—C8'	113.14 (15)	C10—C9—H9A	119.9
C6—N1—C2	121.73 (18)	C8—C9—H9A	119.9
C6—N1—C2'	120.30 (17)	C9—C10—C11	119.7 (2)
C2—N1—C2'	117.74 (17)	C9—C10—H10A	120.2
C2—N3—C4	127.16 (19)	C11—C10—H10A	120.2
C2—N3—H3A	116.4	C9'—C10'—C11'	110.13 (18)
C4—N3—H3A	116.4	C9'—C10'—H10'A	109.6
O1''—C1''—C8'	107.92 (17)	C11'—C10'—H10'A	109.6
O1''—C1''—H1''A	110.1	C9'—C10'—H10'B	109.6
C8'—C1''—H1''A	110.1	C11'—C10'—H10'B	109.6
O1''—C1''—H1''B	110.1	H10'A—C10'—H10'B	108.1
C8'—C1''—H1''B	110.1	C6'—C11'—C10'	112.58 (17)
H1''A—C1''—H1''B	108.4	C6'—C11'—H11'A	109.1
O2—C2—N3	122.6 (2)	C10'—C11'—H11'A	109.1
O2—C2—N1	123.0 (2)	C6'—C11'—H11'B	109.1
N3—C2—N1	114.42 (19)	C10'—C11'—H11'B	109.1
O1'—C2'—N1	105.77 (16)	H11'A—C11'—H11'B	107.8
O1'—C2'—C3'	111.57 (17)	C12—C11—C10	119.9 (2)
N1—C2'—C3'	112.05 (17)	C12—C11—H11A	120.0
O1'—C2'—H2'A	109.1	C10—C11—H11A	120.0
N1—C2'—H2'A	109.1	C11—C12—C7	121.6 (2)
C3'—C2'—H2'A	109.1	C11—C12—H12A	119.2
C2'—C3'—C4'	109.02 (18)	C7—C12—H12A	119.2
C2'—C3'—H3'A	109.9	C18—C13—C14	116.9 (2)
C4'—C3'—H3'A	109.9	C18—C13—Si	120.88 (17)
C2'—C3'—H3'B	109.9	C14—C13—Si	122.19 (17)
C4'—C3'—H3'B	109.9	C15—C14—C13	121.9 (2)
H3'A—C3'—H3'B	108.3	C15—C14—H14A	119.0
C5'—C4'—C3'	109.21 (17)	C13—C14—H14A	119.0
C5'—C4'—H4'A	109.8	C16—C15—C14	119.1 (2)
C3'—C4'—H4'A	109.8	C16—C15—H15A	120.5
C5'—C4'—H4'B	109.8	C14—C15—H15A	120.5
C3'—C4'—H4'B	109.8	C17—C16—C15	120.7 (2)
H4'A—C4'—H4'B	108.3	C17—C16—H16A	119.7
O4—C4—N3	120.0 (2)	C15—C16—H16A	119.7
O4—C4—C5	125.8 (2)	C16—C17—C18	119.6 (2)
N3—C4—C5	114.18 (19)	C16—C17—H17A	120.2
C6—C5—C4	120.3 (2)	C18—C17—H17A	120.2
C6—C5—H5A	119.9	C17—C18—C13	121.8 (2)
C4—C5—H5A	119.9	C17—C18—H18A	119.1
C6'—C5'—C4'	111.68 (17)	C13—C18—H18A	119.1
C6'—C5'—H5'A	109.3	C22—C19—H19A	109.5
C4'—C5'—H5'A	109.3	C22—C19—H19B	109.5
C6'—C5'—H5'B	109.3	H19A—C19—H19B	109.5
C4'—C5'—H5'B	109.3	C22—C19—H19C	109.5
H5'A—C5'—H5'B	107.9	H19A—C19—H19C	109.5
O7'—C6'—O1'	109.20 (16)	H19B—C19—H19C	109.5
O7'—C6'—C11'	111.75 (17)	C22—C20—H20A	109.5
O1'—C6'—C11'	106.17 (16)	C22—C20—H20B	109.5
O7'—C6'—C5'	106.73 (16)	H20A—C20—H20B	109.5
O1'—C6'—C5'	110.88 (17)	C22—C20—H20C	109.5
C11'—C6'—C5'	112.14 (17)	H20A—C20—H20C	109.5
C5—C6—N1	122.0 (2)	H20B—C20—H20C	109.5
C5—C6—H6A	119.0	C20—C22—C23	108.30 (19)
N1—C6—H6A	119.0	C20—C22—C19	108.97 (19)
C8—C7—C12	116.7 (2)	C23—C22—C19	109.75 (18)
C8—C7—Si	121.68 (17)	C20—C22—Si	110.46 (14)
C12—C7—Si	121.27 (16)	C23—C22—Si	111.55 (15)
C9—C8—C7	121.8 (2)	C19—C22—Si	107.78 (15)
C9—C8—H8A	119.1	C22—C23—H23A	109.5
C7—C8—H8A	119.1	C22—C23—H23B	109.5
O7'—C8'—C1''	105.03 (16)	H23A—C23—H23B	109.5
O7'—C8'—C9'	110.61 (17)	C22—C23—H23C	109.5
C1''—C8'—C9'	114.08 (19)	H23A—C23—H23C	109.5
O7'—C8'—H8'A	109.0	H23B—C23—H23C	109.5
C1''—C8'—H8'A	109.0		

### Hydrogen-bond geometry (Å, °)

**Table:**

*D*—H···*A*	*D*—H	H···*A*	*D*···*A*	*D*—H···*A*
N3—H3A···O4^i^	0.86	2.03	2.873 (2)	166

Symmetry codes: (i) −*x*, −*y*+2, −*z*.
